# Social media use to improve communication on children and adolescent’s health: the role of the Italian Paediatric Society influencers

**DOI:** 10.1186/s13052-021-01111-7

**Published:** 2021-08-11

**Authors:** Elena Bozzola, Anna Maria Staiano, Giulia Spina, Nicola Zamperini, Francesco Marino, Marco Roversi, Giovanni Corsello, The Italian Paediatric Society Executive Board, Alberto Villani, Rino Agostiniani, Luigi Memo, Diego Peroni, Giuseppe Banderali, Renato Turra, Nicola Romeo, Alberto Chiara, Dal Vecchio Antonio, Luciana Indinnimeo, Pietro Ferrara

**Affiliations:** The Italian Paediatric Society, via Gioberti 60, Rome, Italy

**Keywords:** Social media, Communication, Health, Influencer, Children

## Abstract

**Background:**

Fake news on children’s and adolescent health are spreading. Internet availability and decreasing costs of media devices are contributing to an easy access to technology by families. Public health organizations are working to contrast misinformation and promote scientific communication. In this context, a new form of communication is emerging social media influencers. Aim of this study is to evaluate the role of paediatric influencers (PI) in communicating information about children and adolescents’ health.

**Materials and methods:**

A group of PI was enrolled from December 2019 to January 2020 by a scientific commission nominated by the Italian Paediatric Society (SIP). PI were asked to share Facebook messages from the official page of the SIP to their own network. Social media tools have been evaluated across 12 months, from July 28, 2019, to July 11, 2020. For the purposes of clarity, we schematically divided the study period as follows: the period of PIs activity (January 6, 2020, to July 11, 2020) and the period when PIs were not yet active (July 28, 2019, to January 4, 2020). Information on Facebook page (lifetime total likes, daily new likes, daily page engaged, daily total reach) and on published post (lifetime post total reach, lifetime post organic reach, lifetime engaged users) were evaluated.

**Results:**

A significant increase in Facebook daily new likes, page engagement and total reach, as well as in lifetime post total and organic reach was evidenced. As for PI, they reported a positive experience in most cases.

**Discussion:**

In the digital era, communication strategies are becoming more important, so that the scientific community has to be actively involved in social media communication. Our pilot study demonstrated that the recruitment of paediatric influencers has increased communication and interaction of the SIP Facebook page.

**Conclusion:**

Our study shows the potential role of influencers: spreading health messages via PI seems to be a successful strategy to promote correct communication about children’s and adolescents’ health.

## Background

The use of the internet has globally increased in the last years. More specifically, an increase from 55 to 86% of internet use has been documented in the last 11 years [1]. Similar to traditional media, such as television and radio, the new social media are becoming day by day more influential in the promotion of products and services. Social media influencers are also an attractive platform to promote public health and their use by health-related organizations is gradually increasing [2]. Promotional campaigns on diabetes, bullying, breast cancer, influenza, vaccinations, tobacco and obesity are spreading through social media. The results are encouraging as a significant gain in health may be achieved. For instance, the successful campaign “The Heart Truth”, aimed at raising awareness among women about heart disease, led to a reduction of heart disease rates [3, 4].

Social media can actively and directly interact with their public, thus overcoming an obsolete one-way communication. This has been proposed as the crucial new concept of social media ecosystem [5–10]. The use of social media in the field of health communication and promotion has substantially increased in the last 10 years. Facebook, in Italy, is particularly interesting when it comes down to paediatric health promotion. According to Facebook Audience Insights Data (available on the Facebook advertising tool), more of the 60% of the almost 30 million Facebook users in Italy are between 25 and 55 years old. On social media, people can be engaged and reached with catchy pieces of content, like infographics, videos, that must be, at the same time, verified and reliable. For people who want to know more, the same information with a different format - i.e. articles – is made available on the website.

In this setting the online influencer, namely a new form of media celebrity, is emerging. Influencers have been recently defined as “individuals on social media who have built a credible reputation and following, oftentimes in a specific topic area” [11]. Their online contributions, opinions and posts can affect their fanbase, thus conditioning public behaviors [12–15]. For such reasons, public health organizations are increasingly encouraging scientists’ active participation in social media communication [9, 10].

The Italian Paediatric Society (SIP) has recently nominated a scientific commission to promote paediatric communication among scientists and families. In the last months, this commission has promoted campaigns on vaccination policy and media use in children, reaching more than 30.000 families via social media. The use of influencers has the potential of reaching a larger audience either directly or through their online contacts. Nevertheless, few studies investigated the effects of the social media influence on promotional campaigns.

Aim of the study is to investigate the role of paediatric influencers (PI) in spreading messages about children’s health and their efficacy in reaching the families.

## Material and methods

A group of PI was enrolled from December 2019 to January 2020 by the Italian Paediatric Scientific Commission. Inclusion criteria were to be a paediatricians, to be self-confident in media usage, to have more than 500 followers or Facebook contacts and to belong to SIP. Each included subject underwent a one-day advanced communication training, performed by communication strategies and marketing experts.

PI were asked to share Facebook messages from the official Facebook page of SIP, including the link to the specific campaign or post.

To verify the efficacy of this communication strategy, social media coverage was evaluated across 12 months, from July 28, 2019, to July 11, 2020. For the purposes of clarity, we schematically divided the study period as follows: the period of PIs activity (January 6, 2020, to July 11, 2020) and the period when PIs were not yet active (July 28, 2019, to January 4, 2020).

We took into account the following variables:

- Lifetime total likes (the total number of people who liked the Facebook page)
- Daily new likes (the number of new people who daily liked the Facebook page)
- Daily page engaged users (the number of people who engaged with the Facebook page, considering engagement any click, or story created)
- Daily total reach (the number of people who viewed any content from the Facebook page).

Information about the published posts was also considered. In details:

- Lifetimes post total reach (the number of people who viewed the single posts, including statuses, photos, links, videos and more)
- Lifetimes post organic reach (the number of people who viewed the single posts through unpaid distribution)
- Lifetime engaged users (the number of people who engaged in certain ways with the page posts, for example by commenting on, liking, sharing, or clicking upon particular elements of the post)

We conducted two different analyses.

Firstly, we compared the data gathered on the variables “Lifetime total likes”, “Daily new likes”, “Daily page engaged users”, and “Daily total reach” during the period of PIs activity (January 6, 2020, to July 11, 2020) to the data on the same variables gathered during the period when the PIs were not yet active (July 28, 2019, to January 4, 2020). We called this “long time frame” analysis.

Secondly, we compared the average change of the three variables “Daily new likes”, “Daily page engaged users” and “Daily total reach” on the day when the PIs shared their posts and the following day, to the average change of the same variables during the period of PIs activity (January 6, 2020, to July 11, 2020). We called this “short time frame” analysis.

Then, we reported data on the variables “Lifetime post total reach”, “Lifetime post organic reach”, and “Lifetime engaged users” related to the posts of the Italian Society of Paediatrics Facebook page shared by the PIs group and compared it to the same data on all other posts, during the period of PIs activity (January 6, 2020, to July 11, 2020).

Finally, an anonymous questionnaire was also sent to the recruited group of PI to evaluate their personal experience (Table 1).

**Table 1:** Anonymous questionnaire form

1. Male or female?	
2. How old are you?	
3. Where do you come from (North, Centre or South of Italy)?	
4. How many contacts do you have on Facebook or other social networks?	
5. What is your employment (doctor, resident, researchers, teacher)?	
6. What are the social media that you used mostly, in order of frequency (e.g. Facebook, Instagram, Twitter)?	
7. How much do you use social media for scientific communications on a scale from 1 to 10?	
8. How much do you use social media to communicate with your patients on a scale from 1 to 10?	
9. According to your experience, did you consider communication through social media and the “paediatric influencer” project a useful communication methodology?”	
10. How much did you share the Italian Paediatric Society contents from 1 to 10?	

The study sample was characterized using descriptive statistics: mean values, standard deviation, minimum and maximum values. The reported data were analyzed with the two-sample t-test. A value of *p* < 0.05 was considered statistically significant.

## Results

### Paediatric influencer demographic data

The sample consisted of 20 PI (11 females, 9 males) aged 29 to 48 years (mean = 36.8 +/− SD 5.88), geographically located in the North (*n* = 5), Centre (*n* = 10) and South of Italy (*n* = 5). Most of them used Facebook (55%) and Instagram (25%) to communicate and interact with their public. LinkedIn and Twitter were less used (15 and 10% of the sample, respectively). The PI had a number of followers on their social networks ranging from 500 to 4400.

### Social media affluence data

During the study period, 11 posts were published by the SIP Scientific Commission on Facebook and shared by the PI.

Considering the type of patient family this media reaches, clickers age distribution was the following: 13–17 years (< 1%), 18–24 years (2%), 25–34 years (27%), 35–44 years (42%), 45–54 years (16%), 55–64 years (8%), > 65 years (5%); Female clickers were the majority (81%) compared with male clickers (18%). Italian origin has been the most observed among clickers (98%), followed by UK (0.5%) and Germany (0.31%). Among the Italy regions the distribution of clickers was the following: Rome (74%), followed by Milano (9%), Naples (7%), Palermo (4%), Lamezia Terme (3%), Turin (3%).

According to the aim of the study, at first we performed the “long time frame” analysis.

There was a statistically significant difference in social media affluence before and after the PI engagement in the long time frame, as presented in Table 2A (Table 2A). In particular, up to July the 11th, 2020 the SIP Facebook page received 3223 new likes, significantly higher compared to the previous 6 months in which it received 1971 new likes. During the study period 18.1 new likes were obtained daily, higher than the previously reported 13.5 daily likes. However, this difference was not statistically significant. On the other hand, we observed a statistically significant increase in the mean daily page engaged users and the mean daily total reach during the PI activity (*p* < 0.05).

**Table 2:** Statistical analysis report on the comparison between social media affluence before and after the paediatric influencer group recruitment (long time frame)

	PIs activity	No PIs activity	*p*-value
Sample (days)	188	160	
Lifetime total likes increase			
Total increase	3223	1962	
Mean increase	17.1	12.3	
Daily new likes			
Mean	18.1	13.5	0.023
Median (IQR)	9 (14)	7 (10)	
Min	1	1	
Max	176	173	
Daily page engaged users			
Mean	755	281	< 0.001
Median (IQR)	292.5 (646)	104.5 (252)	
Min	6	5	
Max	17,548	7144	
Daily total reach			
Mean	9128	4355	< 0.001
Median (IQR)	5327 (9970)	2374 (4389)	
Min	323	227	
Max	98,034	79,664	

**Fig. 1:**
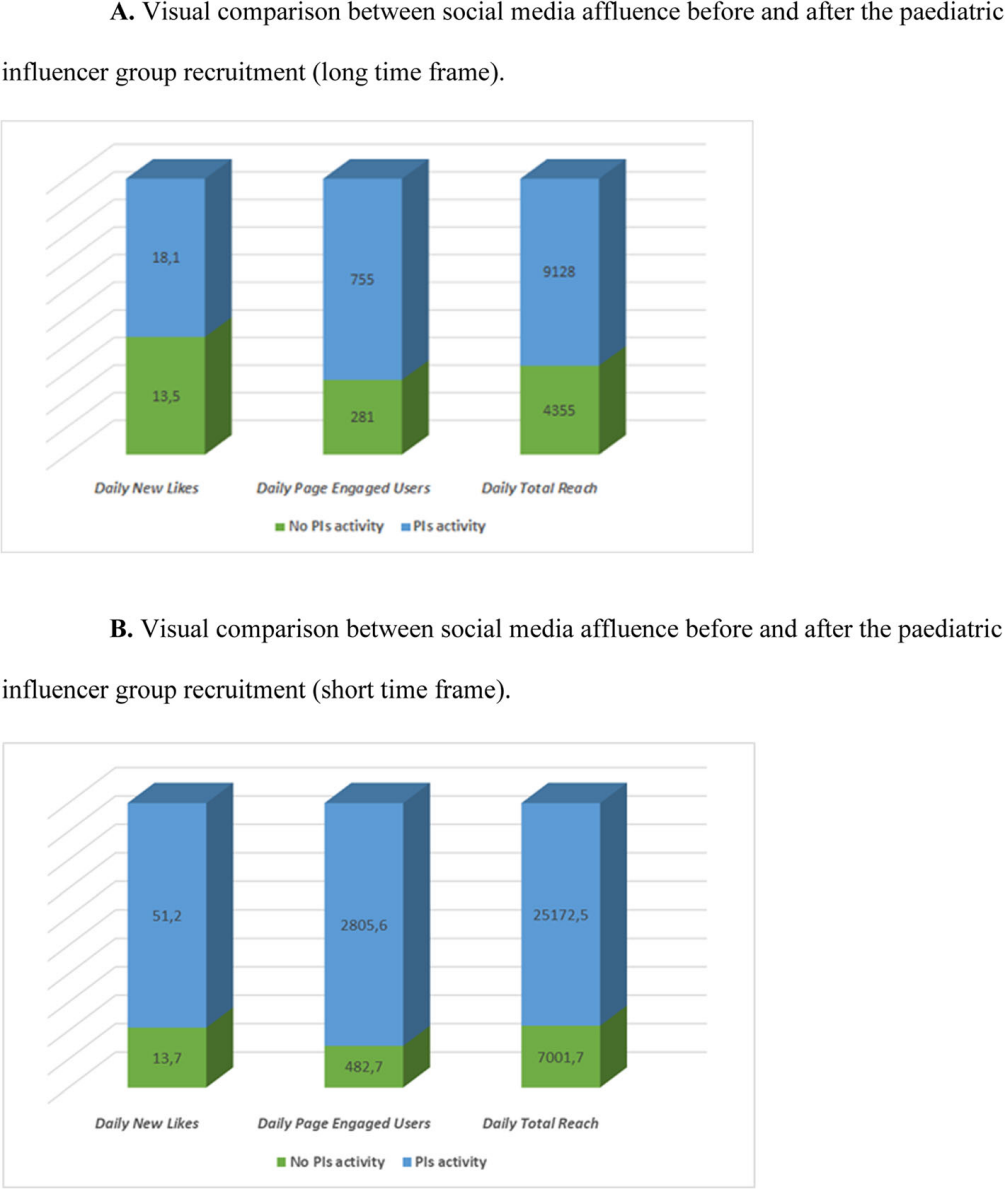
**A** Visual comparison between social media affluence before and after the paediatric influencer group recruitment (long time frame). **B** Visual comparison between social media affluence before and after the paediatric influencer group recruitment (short time frame)

When considering the “short time frame”, we observed a significant increase in daily new likes and daily total reach, with no significant change of daily page engaged users, as reported in Table 2B (Table 2B). Tables 2 and 3 and Fig. 1A and B summarize the results.

**Table 3:** Statistical analysis report on the comparison between social media affluence before and after the paediatric influencer group recruitment (short time frame)

	PIs activity	No PIs activity	*p*-value
Sample (days)	22	166	
Daily new likes			
Mean	51.2	13.7	< 0.001
Median (IQR)	36 (59)	8 (12)	
Min	6	1	
Max	176	151	
Daily page engaged users			
Mean	2805.6	482.7	< 0.001
Median (IQR)	988 (1325)	236 (461)	
Min	59	6	
Max	17,548	8523	
Daily total reach			
Mean	25,172.5	7001.7	< 0.001
Median (IQR)	16,081 (20064)	4382 (7223)	
Min	1847	323	
Max	98,357	58,338

Then, considering contents about posts published during the study period, we observed a significant statistical difference in the variable “Lifetime post organic reach” before and after the paediatric influencer activity (*p* = 0.02). Overall, the most successful posts published were: “Mask for children: how to use?”; “Covid-19: news from the world”; “Mask for children: fake news”.

Descriptive and analytic data about the posts are shown in Tables4 and 5. In details, the variables “Lifetime post total reach”, “Lifetime post organic reach”, and “Lifetime engaged users” related to the posts of the Italian Society of Paediatrics Facebook page shared by the PIs group and compared it to the same data on all other posts, during the period of PIs activity are summarized. (Tables 4 and 5).

**Table 4:** Description of the Italian Paediatric Society Facebook content posted by the Paediatric Influencer group

Topic	Type	Date	Lifetime post total reach	Lifetime post organic reach	Lifetime engaged users
Covid-19: OMS rules for prevention	Photo	June 2020	6491	6491	187
Mask for children: fake news	Video	June 2020	85,090	85,090	3572
Mask for children: how to use	Photo	May 2020	226,361	135,068	41,746
Covid-19: Sip magazine	Photo	April 2020	17,515	17,515	728
Covid-19: how to organize activities for children during lockdown	Photo	April 2020	29,078	29,078	1920
Covid-19: Sip magazine news	Link	March 2020	6297	6297	599
When to go to the Emergency Department in the Covid-19 era	Link	February 2020	9324	9324	1364
Happy Valentine’s Day	Video	February 2020	42,969	42,969	2294
World bullying day	Photo	February 2020	19,894	19,894	906
Covid-19: 5 rules for prevention	Video	February 2020	31,577	31,577	1324
Covid-19: news from the world	Photo	January 2020	112,766	112,766	13,244

**Table 5:** Comparison between parameters of posts published by PI group and other posts from the Italian Society of Paediatrics Facebook page

	Under PI activity	No PI activity	*t*	*p*
Sample (posts)	11	112		
Lifetime Post Total Reach				
Mean	53,396.6	9253.2	2.19	0.53
SD	66,628	14,627.4		
Min	6297	1716		
Max	226,361	131,010		
Lifetime Post Organic Reach				
Mean	45,097.2	8259.2	2.69	0.02
SD	45,153.9	11,149.3		
Min	6297	1716		
Max	135,068	96,451		
Lifetime Engaged Users				
Mean	6171.3	637.6	1.48	0.168
SD	12,354.2	1023.6		
Min	187	20		
Max	41,746	5687		

Finally, by an anonymous questionnaire, PI reported a positive experience in most cases (90%). The questionnaire answers are reported in Table 6.

**Table 6:** PI anonymous questionnaire results

Gender	Number of answers
Male n (%)	9 (45)
Female n (%)	11 (55)
Age	
Mean (range)	36.8 (29–48)
SD	5.88
Geographic origin	
North, n(%)	5 (25)
Centre, n (%)	10 (50)
South, n (%)	5 (25)
Social Media Contacts (range)	500–4400
PI employment	
Medical Doctor, n(%)	13 (65)
Researcher, n (%)	2 (10)
Resident, n (%)	5 (25)
Social Media Used	
Facebook	11 (55)
Instagram	5 (25)
LinkedIn	3 (15)
Twitter	2 (10)
Social media usage for scientific communication (1 to 10 score)	
≤ 6 points, n(%)	0 (0)
7 points, n(%)	2 (10)
8 points, n(%)	10 (50)
9 points, n (%)	5 (25)
10 points, n (%)	3 (15)
Social media usage to communicate with patients (1 to 10 score)	
≤ 5 points, n(%)	0 (0)
6 points, n (%)	2 (10)
7 points, n (%)	3 (15)
8 points, n (%)	10 (50)
9 points, n(%)	3 (15)
10 points, n(%)	2 (10)
PIs feeling about the project: positive or negative	
Positive, n (%)	18 (90)
Negative, n(%)	2 (10)
PIs accordance with the Italian Paediatric Society contents (1 to 10 score)	
≤ 8	0 (0)
9 points, n(%)	2 (10)
10 points, n(%)	18 (90)

## Discussion

The SIP provides high quality topics about Paediatrics and Health care to discuss actuality and new Research published in order to promote health care professional training about Paediatrics issues and Scientific Research and communication. We recognized that the recruitment of paediatric influencers has increased communication and interaction of the Italian Paediatric Society Facebook page (https://www.facebook.com/societaitalianadipediatria). In particular, we documented that a correct use of social media platforms can be a great instrument to spread information. A limit of the study is that we did not analyze the effect of pediatricians’ actions on the health of children and adolescents. We focused on the potential role of influencers in communication. Our results are similar to those of other social campaigns promoted, such as a big campaign conducted in the USA to inform about flu vaccination using influencers and a social media influencer marketing campaign about children’s food intake. The studies reporting on this campaign proved the positive of social media influencers on health promotion [16, 17]. Moreover, a UK Survey conducted to investigate health care professional opinions on social media demonstrated that a percentage of 73.3% of responders considered social media as a negative instrument of communication [18]. Our experience is in contrast with this report. In order to overcome negative experiences of clinicians with social media communication, it is important to follow specific guidelines and criteria [18]. Otherwise, strategies for social media and web presence should be applied. For example, some reports described the “best times to post content in social media” considering 1–4 pm and 2–5 pm as the best hours to post and 1–2 times a day the best frequency to post [19]. In contrast, our social media strategy did not follow any specific time or frequency to post, pointing out the same efficacy and strength. Indeed, the paediatric influencer group used to post the contents suggested at the time and frequency they preferred.

Our project has been involved in the implementation of social media communication through a group of medical doctors concerning paediatrics topics. The simple size of PI was small as it was a pilot study, conducted to verify the usefulness of their involvement in scientific communication. To our knowledge, this is one of the first Italian studies investigating this kind of strategy directly involving clinicians. Some Researchers recommended the creation of a network between clinicians and social media influencers and with “mommy blogs” [20, 21]. Moreover, a Netherlands study conducted on vaccine hesitancy demonstrated how social media influencers can be considered as partners for vaccination and health strategies [22]. Our study is in line with these findings, considering influencers as an instrument to implement health communication [23]. Conversely, our project defined and studied as influencers a group of medical doctors skilled on Paediatrics and communication strategies, documenting a potential role in health promotion to combat medical misinformation.

Pediatric influencers were asked to disseminate validated messages performed by the Italian Pediatric Society, which represents the filter of the information spread and may certify the validity and trustiness of the material. In this way, young and Internet-savvy pediatricians contributed to the spread of official and certified messages on health. In the digital era, the scientific community has to be actively involved in social media communication. Our data revealed that a percentage of 90% of clinicians participating in the study used social media as a communication tool: Facebook has been the most common choice among participants (55%), followed by Instagram (25%) and LinkedIn (15%). These observations are in line with literature showing an increasing frequency of social media usage for scientific communication among clinicians [24–26]. On the contrary, previous studies showed that a percentage of 73% of involved researchers declared to never use social media for scientific communication [27].

The important role of PI in sharing information is mainly evident analyzing the short time frame. As well as in our study, recent reports underline the role of medical social influencers in facilitating the dissemination of information to rapidly reach a larger audience and deliver health care information. The successful dissemination of health-related information by social networks depends on effective dialogic communication and interpersonal influence that facilitate the sharing of information with the public. As well as in our study, previous researchers demonstrate the role of medical social influencers in terms of the number of likes, comments, and especially shares of contents [28]. In the USA, social campaigns on flu vaccination and alimentation using influencers highlighted the positive role of social media influencers for health promotion about children’s food intake [16, 17]. Moreover, sharing information as a form of engagement, which enables users to link the medical social influencer post to their social group have been help increasing trust and disseminate health-related information more widely and rapidly [29].

The efficacy of this communication process may be enforced if medical health influencers are trained by communication specialists on the choice of social media profile - personal or professional –and on the engaging strategy to increase the popularity of the posts. For example, health information communicated through photos and videos may be more appreciated and facilitate the public perception that the information is useful, while hashtags enable the users to share important topics in social media conversations [30, 31].

Of note, there is an increasing number of users that try to obtain health-related information on social network. As a consequence, it is important to provide an interactive communication and a dialogue with the followers to answers relative questions but avoiding substituting a direct medical clinical visit of the patient [32].

Considering contents shared on social media, our results showed the most successful post to be about “health education”, specifically on Covid-19 (“Mask for children: how to use?”, see Table 4). Conversely, some researchers documented that the posts concerning personal stories more than posts about health and education have been the most appreciated having a greater number of total reaches per post [33]. For that purpose, we aimed at providing more posts about personal stories.

Day by day, communication strategies are becoming more important, so that paediatricians should consider social media algorithms to optimize the content for any social media platform. The Facebook algorithm controls the ordering and presentation of posts. Rather than publish content chronologically, posts are presented based on what Facebook sees as relevant to the user. Everything we see in Facebook’s homepage - the so-called Newsfeed - is controlled by an algorithm that takes into account several factors in order to establish which post to show first to each user. Communication experts partially know how to deal with this algorithm even if nobody exactly understand how it works. This mean we can never be completely sure of how many people will see a determined post we decide to publish on a Facebook page. As suggested by communicators, creating and sharing great contents, adapting them to the audience, posting frequently and promoting interactions may be good strategies to face the problem. As a consequence, it is important that PI should be trained on social media algorithms in order to increase post shareability. In our experience, the recruitment of paediatric influencers increased interaction with the SIP Facebook page, and favored the communication of health messages to the children’s’ caregivers. In this scenario, the effective use of social media platforms helps to convey correct scientific information, while contrasting fake news. Using PI to spread health messages proved to be a successful strategy in spreading health messages, likely because influencers were all medical doctors. According to a UK survey, the health care professionals’ opinion on social media as a means of communication was overall negative [18]. In our study, 90% of the PI reported a positive experience as for their engagement. In order to overcome negative experiences of clinicians with social media communication, it is important to follow specific guidelines and criteria, such as the use of privacy setting, separating personal and professional content online [34]. Our study has a main limitation. As the study period overlapped with the first wave of Covid-19 pandemic in Italy, this may be a potential confounder in increasing the use of social media among the users. Anyway, we considered the Engagement Rate (ER) to assess the efficacy of a post or of a Facebook Page, which may be calculated as the number of Engaged Users divided by the total reach of that post, multiplied by 100 to turn it into a percentage. Comparing our results with the average ER on Facebook for posts of any kind during first months of 2020, we can note PI activity efficacy in spreading medical information. In fact, we found out a better ER with PI involvment. In the long time frame, the SIP Facebook page without PI had an average of 6.45% ER and an average 8.27% with PI involvement. In the short time frame, SIP Facebook page had an average 6.89% ER without PI and an average 11.15% with PI. It as an important result, considering that, according to one of the most famous digital metrics’ report, due to lockdown, the average ER on Facebook for posts of any kind during first months of 2020 went up to 3.41%, with just a growth of 0.7% compared to the last quarter of 2019 [35].

## Conclusion

In conclusion, we would like to underline the potential application of influencers involvement in communication strategy. This study highlights the positive impact of social media in promoting communication about children’s and adolescents’ health. In particular, engaging paediatricians as influencers seems to be a valid strategy to improve health communication in the field of paediatrics. However, any medical doctor and health care professional may promote health messages among patients and families, achieving two significant results in one shot: spreading correct information and contrasting the diffusion of fake news on sensible health topics.

## Data Availability

Availability of data and material at dr Bozzola’s repository.

## Abbreviations

PI: Paediatric influencer
SIP: The Italian Paediatric Society
